# Local Function Conservation in Sequence and Structure Space

**DOI:** 10.1371/journal.pcbi.1000105

**Published:** 2008-07-04

**Authors:** Nils Weinhold, Oliver Sander, Francisco S. Domingues, Thomas Lengauer, Ingolf Sommer

**Affiliations:** Max Planck Institute for Informatics, Saarbrücken, Germany; Columbia University, United States of America

## Abstract

We assess the variability of protein function in protein sequence and structure space. Various regions in this space exhibit considerable difference in the local conservation of molecular function. We analyze and capture local function conservation by means of logistic curves. Based on this analysis, we propose a method for predicting molecular function of a query protein with known structure but unknown function. The prediction method is rigorously assessed and compared with a previously published function predictor. Furthermore, we apply the method to 500 functionally unannotated PDB structures and discuss selected examples. The proposed approach provides a simple yet consistent statistical model for the complex relations between protein sequence, structure, and function. The GOdot method is available online (http://godot.bioinf.mpi-inf.mpg.de).

## Author Summary

Proteins are an essential class of molecules playing a variety of roles within a cell. They can be described in various ways: amongst others, by sequence, structure, and function. Determining protein function by wet lab procedures is challenging and tedious. Simultaneously, sequencing and structural genomics projects turn out ever increasing numbers of protein sequences and structures, which are largely lacking functional characterization. As a consequence, there is a growing demand for computational methods that can assist human experts in the functional annotation of proteins. We present a method for protein function prediction based on a novel concept, called local function conservation. Local function conservation in sequence and structure is determined by rigorously analyzing the variability of protein function with respect to sequence and structure similarity. Our method predicts protein function even if the protein to be functionally annotated has only distant relatives. Furthermore, we estimate the reliability of the function prediction. With this approach, we advance automated function prediction and contribute to a better understanding of the complex relations between protein sequence, structure, and function.

## Introduction

Protein structure databases are growing at a rapid rate and, in recent years, structural genomics initiatives have increased the growth rate further. Yet many protein structures remain without functional annotations. Low coverage of functional annotations substantiates the necessity of reliable automated methods for predicting the functions of proteins.

A widely accepted vocabulary for characterizing gene and protein function is maintained by the Gene Ontology (GO) Consortium [1]. To understand protein function, information is typically inferred from evolutionarily related proteins. Evolutionary relation can be determined by sequence similarity. Enzymes, for example, tend to have a conserved function, when they share more than 40%–50% sequence identity [2]–[4]. Inference according to only sequence similarity is not very reliable for accurate function prediction, in particular for remote homology [5],[6].

Some function prediction methods transfer function from similar sequences, such as GOtcha [7], Blast2GO [8], or PFP [9]. Phylogenomic methods, such as SIFTER [10] and Orthostrapper [11], additionally consider knowledge on the evolution of homologous proteins. Motif databases, such as EMOTIF [12], PROSITE [13], and PINTS [14] are used to extract functionally relevant signatures of proteins. Gene3D [15] compiles Hidden Markov Model signatures for CATH families and links these signatures to GO functions. FSSA [16] and PHUNCTIONER [17] use structural signatures derived from proteins of similar function to predict molecular function of uncharacterized proteins. Some approaches use different types of structural features to predict function [18],[19]. Other methods employ sequence-derived protein features [20], genomic context [21], and GO term co-occurrence [22]. Some approaches to function prediction combine several features derived from the protein, or combine predictions from different methods [23]–[25]. Two recent reviews [18],[26] provide an overview of state-of-the-art predictors and discuss many of the aforementioned methods in detail.

The underlying idea of similarity based function transfer is that proteins with similar sequence and structural features are likely to perform the same function [27]–[29]. We take this principle one step further by examining groups of similar proteins. Such a group can be seen as a local region within the protein universe. A molecular function that is shared by all proteins in a local region is considered to be conserved. Local regions may be interspersed with proteins not annotated with this function and function conservation can vary between different regions [30],[31]. Therefore, we use the frequency of functionally identical proteins within a local region to determine the extent to which a function is conserved in the respective region of protein space. The degree of local function conservation is regarded as a confidence measure for the prediction, high conservation implying high confidence that the respective function is correct. This quantitative estimate yields a differentiated view on function conservation, enabling us to predict protein molecular function more accurately.

## Results/Discussion

We estimate the rate of errors made when inferring protein function annotations based on protein sequence and structure similarity. On a representative set of protein domains, the error rates when inferring function naively are considerable. Additionally we analyze how inference is potentially improved by combining different measures for protein similarity.

Within the space spanned by the set of representative protein domains, we identify regions where function is locally conserved. The information how well a molecular function is conserved in a protein neighborhood is captured and used for predicting protein function for new proteins falling into that neighborhood. The prediction method is extensively assessed and we compare its performance with the published PHUNCTIONER method [17]. Finally, we apply the method to 500 uncharacterized structural genomics targets from the PDB and discuss some of the findings in detail.

### Error Rates for Function Inference

The analysis is based on a set of 7290 representative protein domains with maximal 40% sequence identity as provided by the ASTRAL Compendium [32]. Molecular function annotations for the proteins were taken from the Gene Ontology Annotation (GOA) Project [33],[34] (see Methods for details).

Of the 7290 representative protein domains, 86% are annotated with at least one molecular function GO term and 84% are annotated with a molecular function GO term from level three, or more specific (see Methods for the definition of GO levels). Some GOA annotations cannot be resolved to domain precision. Therefore, we reduced the domain set to single domain structures (see Methods for details). Out of this reduced set of 4099 single domain structures, 3449 (84%) domains are annotated with molecular function GO terms. The subsequent analyses are performed on this set of 3449 protein domains. These 3449 protein domains are annotated with 0 to 11 level three GO terms (with a first quartile of 1, a mean of 1.96, and a third quartile of 3 GO terms).

The domains are compared against each other with different measures for protein similarity (see Methods for details): for measuring similarity we use two sequence-based programs, namely local profile alignment (LP) and global profile alignment (GP) [35], and two structure-based programs, namely Combinatorial Extension (CE) [36] and TM-align (TM) [37].

How reliably can functional annotations be inferred from the neighboring proteins of a protein according to each similarity measure? This question is analyzed for GO level three. With a leave-one-out cross-validation for each protein we assess the errors made when inferring GO terms from the nearest neighbor to each protein. The average percentage of correct annotation inferences ranges from 51% to 62%, depending on the similarity measure (55% for CE, 51% for TM, 62% for LP, 62% for GP). Compared to other studies [28],[29], we observe slightly lower error rates.

**Figure 1:**
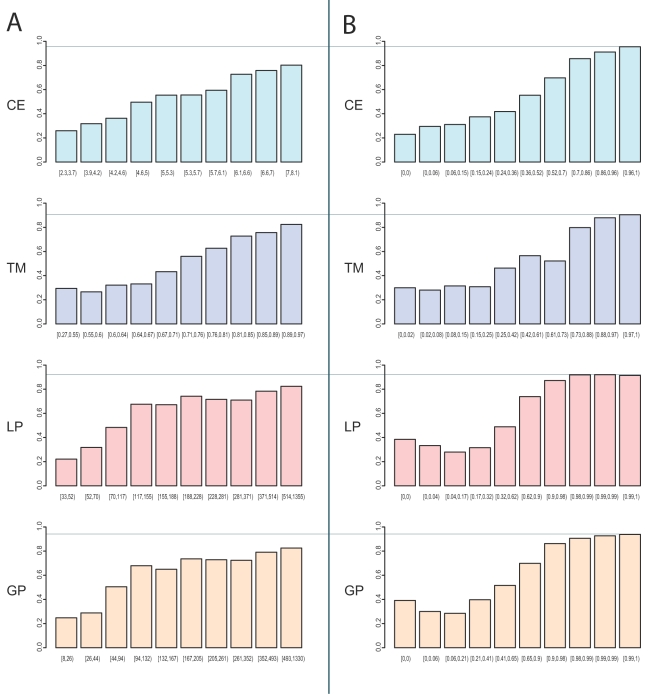
Assessing Similarity-Based Inference. (A) The plot serves to assess the errors made when inferring GO terms from the nearest neighbor of each protein. The inferred annotations are sorted according to the similarity measures (CE, TM, LP, GP) and then binned such that each bin contains an equal number of annotation counts (ca. 670 annotations). This allows for comparing the number of errors for the inference according to different similarity measures which are operating on different scales. The x-axis denotes the range of similarity measure scores falling into that bin, the y-axis the ratio of correct annotations in that range. (B) In contrast to (A), the inferred annotations are sorted according to raw function conservation scores, based on the similarity measures (CE, TM, LP, GP). The x-axis denotes the range of raw function conservation scores falling into that bin, the y-axis the ratio of correct annotations in that range.

In Figure 1A the inferred annotations are sorted according to the similarity measures and then binned such that each bin contains an equal number of counts (ca. 670 annotations). This allows for comparing the number of errors in inference according to different similarity measures, where the different similarity measures are operating at different scales. Even for very similar proteins, in the highest scoring bins, we observe a maximum of only 83% annotations being correctly inferred. Consequently, when inferring annotations from nearest neighbors without further analysis, at least 17% of the annotations are predicted falsely. The situation is even worse for lower similarity ranges. These errors can be attributed to the local properties in sequence and structure space. They demonstrate the difficulty of function annotation transfers at different similarity ranges.

### Combining Similarity Measures for Sequence and Structure

**Figure 2:**
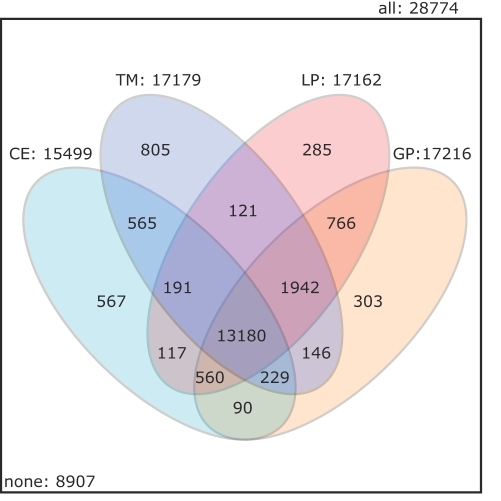
Impact of Different Similarity Measures on Inferring Function. The four-set Venn diagram covers the correct GO term inferred from the neighbors based on the individual similarity measures. Each ellipse represents the number of GO terms correctly inferred using one similarity measure. The numbers of GO terms correctly inferred by several similarity measures are shown in the intersections between one or more ellipses.

We broaden the above analysis to all GO levels, and examine to which extent function prediction can potentially benefit from combinations of protein similarity measures. The Venn diagram in Figure 2 shows how the set of GO annotations decomposes into subsets that can be inferred from protein neighbors according to different similarity measures. Altogether, there are 1806 distinct GO terms attached to 3449 proteins, yielding 28774 annotations. Of these, 8907 annotations are not found at a nearest neighbor according to any similarity measure. The remaining 19867 GO annotations are found at the nearest neighbor according to at least one similarity measure.

The numbers of annotations that could be inferred by one similarity measure alone range from 15499 (53% for CE) to 17216 (60% for GP). Thus, if only one of the similarity measures were used for function inference, one would miss between 2651 (9%) and 4368 (15%) correct annotations that could be inferred using all four similarity measures. The diagram demonstrates clearly that there is potential in the combination of several similarity measures for predicting GO terms.

### GOdot: Using Local Function Conservation for Predicting Molecular Function

In the previous sections, we demonstrated that inferring function according to annotations attached to the nearest neighbors is useful but prone to errors. We also showed that combining different similarity measures yields a potentially better coverage of predicted GO terms. Here, we propose the GOdot method which combines the information from several similarity measures and assesses local function conservation in protein sequence and structure space in order to predict GO molecular function.

#### GOdot: method overview

**Figure 3:**
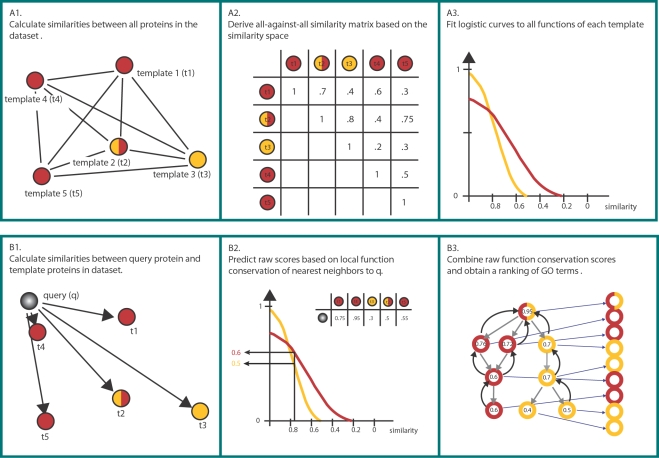
Overview of the GOdot Method. We exemplify the GOdot method on a set of five template proteins (t1–t5) having two different molecular functions (drawn in yellow and red, respectively). The training procedure (top row) consists of similarity calculations (A1), yielding four different similarity matrices one of which is shown (A2). Based on these similarities, logistic curves are fitted for each molecular function in the dataset (A3). The prediction (bottom row) comprises similarity computations between the query protein and the proteins in our dataset (B1), which are then used to predict the conservation of molecular functions in the queries proximity (B2). The final ranking of GO terms is obtained using combination schemes along the GO graph structure (B3). See Methods section for details.

**Figure 4:**
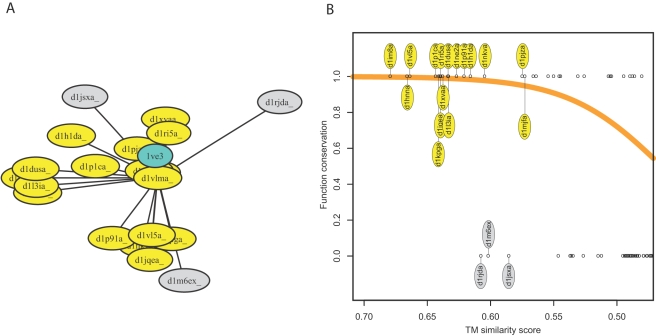
Sample Neighborhood. (A) Using TM to identify the nearest neighbor of the sample query protein 1ve3 yields protein domain d1vlma. For d1vlma the TM scores were pre-computed, resulting in the neighborhood illustrated here with Kruskal's non-metric multidimensional scaling [44](where similar proteins structures are depicted close). Domain d1vlma has several molecular functions attached, for this illustration we selected GO∶0008757 (S-adenosylmethionine-dependent methyltransferase activity). Protein domains having this function are colored yellow, domains not annotated with this function are colored in grey. (B) TM scores with respect to d1vlma are sorted along the x-axis. Protein domains annotated with molecular function GO∶0008757 are assigned a y coordinate of 1 (drawn in yellow), domains not annotated with this function are assigned a y coordinate of 0 (drawn in grey). Unlabeled domains are from the 200 nearest neighbors of d1vlma. A logistic curve is fit through these points (drawn in orange). The logistic curve can be evaluated for the raw function conservation score for a given TM score.

The GOdot method comprises two stages: a training stage which is performed only once, and a prediction stage that is run once for each unknown query protein. The complete protocol is illustrated in Figures 3 and 4 and explained in full detail in the Methods section.

The training is performed on the above-mentioned set of protein domains with no more than 40% sequence identity. Within the space of these proteins, the method looks for regions of similar domains having the same function. The degree of function conservation in such regions can vary considerably depending on the type of molecular function, the number of protein domains having that function, and the metric used to calculate protein similarity. Therefore, we estimate the degree of function conservation separately for each GO molecular function in the region around each protein domain. Analyzing the region of 200 nearest neighbor proteins and using logistic regression, we obtain one logistic curve for each GO term, capturing the extent of functional conservation in the region around the protein domain. The logistic regression is done separately for each similarity measure.

In the prediction phase, the method predicts the molecular functions for an unknown query protein using the pre-computed logistic curves. Initially, the uncharacterized query protein is compared to all protein domains in the training set. The logistic curves of the most similar proteins in the data set are used to predict the molecular functions of the query: if the logistic curve of one of these nearest neighbors indicates high function conservation for a specific GO term in the region of the query, the GO term is predicted with a corresponding raw function conservation score. Raw function conservation scores are deduced from the logistic curves based on the similarity of the query to the nearest neighbors. The method computes several raw conservation scores for the query structure, one for every GO term annotated to the nearest neighbors. Usually, there exist several raw function conservation scores for one and the same GO term, either from different nearest neighbors with the same functions, or from one protein domain selected as nearest neighbor by different similarity measures. In order to assign one score to each of the predicted GO terms, we have developed two alternative schemes of combining raw function conservation scores along the GO graph: selective combination and consensus combination. Both combination schemes ensure that a GO term is predicted together with all its ancestors in the GO hierarchy, and that parental GO terms obtain scores that are at least as high as those of their GO descendants. This approach is in compliance with the GO true path rule, which states that “the pathway from a child term all the way up to its top-level parent(s) must always be true” (http://www.geneontology.org/GO.usage.shtml#truePathRule). The combined scores provide an estimate for the reliability of the predicted GO terms.

#### GOdot: illustration of the method with a sample query

We illustrate the GOdot function prediction mechanism for a sample query protein with PDB ID 1ve3, for which a crystal structure is available from a structural genomics initiative. In the representative set, the nearest neighbors according to CE, TM, LP, and GP are the protein domains d1p91a (CE score 5.9), d1vlma (TM score 0.74), d1vl5a (LP score 188), and d1qama (GP score 136). For each of these, the surrounding space was pre-analyzed. In Figure 4A, the surrounding region is depicted for d1vlma according to TM similarity. The domain d1vlma has molecular functions GO∶0003824, GO∶0008168, GO∶0008757, GO∶0016740, and GO∶0016741 attached. Here, we focus on GO∶0008757 (S-adenosylmethionine -dependent methyltransferase activity). In Figure 4A, protein domains having function GO∶0008757 are colored yellow, domains not annotated with this function are colored grey. The domains with this function form a cluster within which we find the query protein.

Numerically, local function conservation is captured with the raw function conservation score. As depicted in Figure 4B, the neighbors of d1vlma are sorted according to the TM scores with respect to d1vlma, and a logistic curve is fit. Evaluating the logistic curve at a TM score of 0.74 (from 1ve3 to d1vlma), yields a raw function conservation score of 0.9955. Similarly, measuring with CE, LP, and GP, the raw function conservation scores for GO term GO∶0008757 are 0.9817, 0.9980, and 0.9998, respectively. These are computed from the above-mentioned nearest neighbors, which are all annotated with this molecular function.

Using the combination schemes, the raw function conservation scores are combined selectively into 0.9998 (which is the maximum of the above raw function conservation values), and into ≈1–10^−11^ according to the consensus combination (which is 1−(1−0.9817)·(1−0.9955)·(1−0.9980)·(1−0.9998)). For the sake of simplicity, we have not included any GO term of the nearest neighbors more specific than GO∶0008757 into the above calculations.

### Assessment of the GOdot Method

To assess the performance of the GOdot method for function prediction, we compare four variants of function predictors: function inference based on protein similarity alone (as discussed above), function inference based on raw function conservation scores, function inference based on selectively combined function conservation scores, and function inference based on consensus combined function conservation scores.

#### Reliability of raw function conservation scores

Do raw function conservation scores improve the performance when predicting function? In Figure 1A, we sorted the inferred annotations according to similarity measure scores. In Figure 1B, annotations are sorted according to raw function conservation scores. Again, the inferred annotations are binned such that each bin contains an equal number of counts. The two figures (Figure 1A and Figure 1B) are directly comparable. For high raw function conservation scores, the rates of correctly predicted annotations range from 90% to 95% (compared to rates of 80% to 83% in Figure 1A). For low raw function conservation scores, the rates of correctly predicted annotations are below 50%. Compared to Figure 1A, the separation between correct and incorrect function inferences is much better. Consequently, the raw function conservation score adequately reflects the confidence that we have into a prediction.

#### Assessing combined function conservation scores

**Figure 5:**
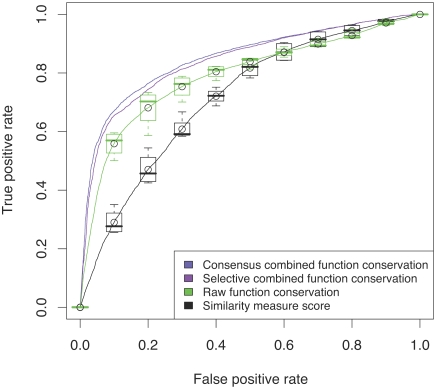
Comparing Similarity Scores to Raw and Combined Function Conservation Scores. The ROC plot serves to analyze the reliability when inferring GO level three functional annotations from the nearest protein neighbors. For each protein domain, nearest neighbors are sought according to the four similarity measures (CE, TM, LP, GP). The GO terms attached to these nearest neighbors can be potentially inferred for a query protein. By sorting annotation transfers according to the similarity scores and evaluating the true positive rate versus the false positive rate, a ROC curve is derived.The black curve displays the average ROC curve for the four similarity measures (CE, TM, LP, GP); the boxplots attached serve to estimate the observed spread. Similarly, when sorting according to raw function conservation scores, we obtain four ROC curves, the average of which is shown as green curve along with the estimated spread as boxplots. Merging the information into a combined consensus score yields one score per inferred annotation; The corresponding ROC curve is plotted in violet for selective combination and in blue for consensus combination.

How good is the quality of function inference based on combined function conservation scores compared to inference based on raw function conservation or to the naive inference based on the similarity measures alone? In Figure 1A the correct and incorrect annotations obtained for a similarity score were assessed with a leave-one-out cross-validation for annotations of GO level three. By thresholding according to the scores and evaluating the true positive rate versus the false positive rate, we produce one ROC curve for each similarity measure. The black curve in Figure 5 displays the average ROC curve for the four similarity measures (CE, TM, LP, GP); the boxplots attached serve to estimate the observed spread. This curve summarizes the four plots in Figure 1A. The average area under the ROC curve (AUC) is 0.71.

Similarly, when sorting according to raw function conservation scores, as in Figure 1B, we obtain four ROC curves, the average of which is shown as green curve along with the estimated spread (AUC 0.79). When merging the information into one combined consensus score, one obtains only one score per inferred annotation and consequently only one ROC curve. In Figure 5, this curve is marked in violet for the selective combination and in blue for the consensus combination. We observe that the combined score outperforms the raw function conservation score, which in turn outperforms the use of similarity measures. The consensus combination (AUC 0.87) outperforms the selective combination (AUC 0.86) slightly (the difference between the combination schemes is significant as we discuss in the Text S1 and Figure S1). The selective combined score is typically identical for the highest scoring GO term and its GO generalizations (as the maximum scores are propagated up along the GO hierarchy, see Methods for details). The score combined by consensus integrates the conservation scores of all GO descendants of a GO term to be scored, producing a more differentiated ranking of GO terms.

Employing the function conservation concept clearly improves the prediction performance. The two GOdot predictors using function conservation scores significantly perform better than the reference predictors.

#### Additional assessment on high-quality annotations

The Gene Ontology Annotation Project (GOA) keeps track of the sources of their functional annotations by use of evidence codes. An overview of the Evidence codes used by GOA is provided in Table 1. Curated function assignments can stem from direct experiments (evidence codes IDA, IEP, IGI, IMP, IPI), literature (TAS, NAS), or computational methods validated manually (ISS, IGC, IGC). GOA collects electronically inferred annotations (IEA) using various computer-based resources (http://www.ebi.ac.uk/GOA/goaHelp.html), such as the Ensembl Compara method [35], or BLAST homology searches with a conservative E-value of 10^−50^ (http://www.geneontology.org/cgi-bin/references.cgi). The exact IEA origin is only tracked for function assignments made after May 2007.

**Table 1:** Evidence Codes Used by the Gene Ontology Annotation Project.

**Curator-Assigned Evidence Codes**		
IDA	Inferred from Direct Assay	Experimental
IPI	Inferred from Physical Interaction	Experimental
IMP	Inferred from Mutant Phenotype	Experimental
IGI	Inferred from Genetic Interaction	Experimental
IEP	Inferred from Expression Pattern	Experimental
ISS	Inferred from Sequence or Structural Similarity	Curated Computational Analysis
IGC	Inferred from Genomic Context	Curated Computational Analysis
RCA	Inferred from Reviewed Computational Analysis	Curated Computational Analysis
TAS	Traceable Author Statement	Author Statement
NAS	Non-traceable Author Statement	Author Statement
IC	Inferred by Curator	Curator Statement
ND	No biological Data available	Curator Statement
**Automatically Assigned Evidence Codes**		
IEA	Inferred from Electronic Annotation	Automatically Assigned

The table lists evidence codes as defined by the Gene Ontology Consortium (http://www.geneontology.org/GO.evidence.shtml). It shows the evidence codes, corresponding phrases, and broader categories describing how the evidence codes are associated with gene products.

On the previously studied set of 3449 representative domains there is the following evidence for function annotations: 8% are based on direct experiments, another 8% are based on literature statements, and less than 1% are found by curators based on computational evidence. The rest (83%) of the annotations is based on automatic electronic inference.

When using these functional annotations for training a new computational method like GOdot, there is an obvious trade-off between quality and coverage: the higher the number of annotated proteins used, the lower is the ratio of manually curated annotations on these. The previously described set strives for maximum coverage, as this is the aim in a typical application scenario. We also tested the method on a second set based on high-quality annotation data.

**Figure 6:**
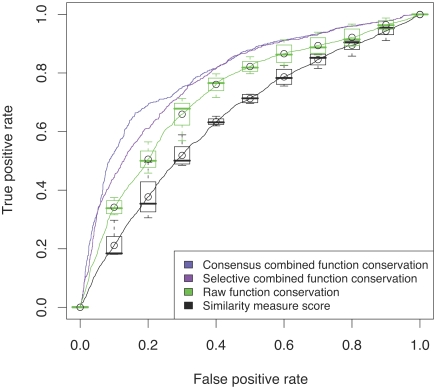
On Experimental Annotation Data Only. Comparing similarity scores to raw and combined function conservation scores. ROC analysis on a reduced high quality data set containing only experimental annotation data (evidence codes IDA, IEP, IGI, IMP, IPI) for 629 proteins. The black curve displays the average ROC curve for the four similarity measures (CE, TM, LP, GP); the boxplots are an estimate of the observed spread. The green curve corresponds to the average of the four raw function conservation scores. The ROC performance of selective and consensus combination is shown with the violet and blue curves, respectively.

The high-quality data set is restricted to annotations that stem traceably form literature (evidence code TAS) or from direct experiments (evidence codes IDA, IEP, IGI, IMP, IPI), leaving 945 proteins with curated experimental annotations. We repeated the analyses described in the previous section on this high-quality subset. The results are summarized in Figure 6 and Figure S2. Compared to the high-coverage data set shown in Figure 5, all performance curves in Figure 6 are lower. Due to the subsampling, nearest neighbors are farther apart and harder to detect, making predictions more difficult. Nevertheless, the same trends are clearly visible: the GOdot raw function conservation scores (AUC 0.73) are better suited for function inference than plain similarity measures (AUC 0.64), and combining the raw function conservation scores further improves the performance (AUC 0.78 and AUC 0.80 for selective and consensus combination). Figure S2 is an analog to Figure 1, but based on the high-quality subset. It outlines the errors of function inference made using similarity measures alone (A) and raw function conservation scores (B).

The results based on high-quality data confirm our observations made on the larger high-coverage data set. Since the higher coverage enables predictions for a broader range of GO functions and a more diverse set of query proteins, we have used it throughout this article. It is important to note that IEA annotations from GOA are based on very closely related proteins with high sequence and structure similarity. GOdot overcomes this limitation by exploiting distant relations of pairwise sequence identities below 40%, thereby going beyond current IEA approaches.

#### Assessment according to the PHUNCTIONER protocol

Finally, we assessed the GOdot method according to the protocol published with the PHUNCTIONER method [17]. The PHUNCTIONER method uses structural multiple alignments of functionally similar proteins to derive position specific scoring matrices (PSSMs) for specific GO functions; structures with unknown function are scanned against a library of PSSMs to assign function to the structure. PHUNCTIONER can predict 121 molecular function GO terms from different levels of the GO hierarchy. For a query protein, it predicts one of these GO terms along with a score. The list of predicted GO terms is sorted according to the scores from likely to unlikely. The assessment of the PHUNCTIONER method was previously performed with ROC plots [17]. These ROC plots are GO-level specific and were constructed as follows. For GO level three, only the highest scoring level-3 GO term from the prediction list is considered for one query and evaluated to be either true or false. Sorting predictions for multiple queries according to their scores one obtains a ROC curve. The PHUNCTIONER method was assessed this way on sets of up to 6168 query proteins, where query proteins were selected such that at least one of their annotated GO terms was predictable by the PHUNCTIONER method. We have repeated this evaluation procedure in an analogous fashion on the set of 3449 protein domains also employed in our other experiments.

**Figure 7:**
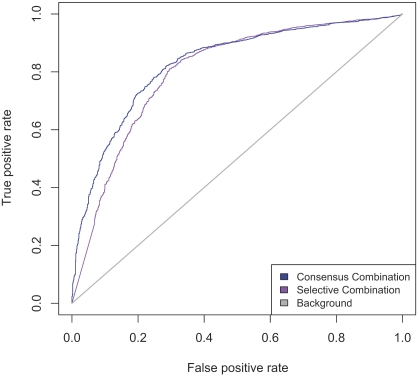
Evaluation According to PHUNCTIONER Protocol. Following the protocol described for evaluation of the PHUNCTIONER method in [17], the ROC curve considers only the highest scoring predicted level three GO term for each query protein. A diagonal line in the ROC plot indicates random predictor performance. Optimal performance is demonstrated by a curve passing through the upper left corner.

The resulting ROC curves for the GOdot method are shown in Figure 7. The two GOdot predictors, using selective and consensus combination of scores, are compared to a baseline reference predictor. The reference predictor predicts GO terms based on their background frequencies within the dataset. The curve of an optimal predictor would pass through the upper left corner of the plot, a diagonal line in the ROC plot indicates random performance. Indeed, the background reference predictor matches the diagonal closely. The GOdot selective and consensus predictors are clearly superior to the reference predictor.

The performance comparison with PHUNCTIONER underlies the following restrictions: (i) Since the PHUNCTIONER method is not available to us we had to use the previously published results [17] on the validation of the method. (ii) Since the benchmark dataset for the published validation of PHUNCTIONER is not accessible to us in the form used for the validation we have to compare the performances of the two methods on differing datasets. Taking this into account, we realized a scenario which makes it harder for GOdot, in principle, to attain the same true positive rate for a given false positive rate. We did so by assessing the performance of GOdot on the full set of 1806 GO terms (compared to only 121 GO terms for PHUNCTIONER). The performance we observe for GOdot is higher than that reported for PHUNCTIONER. As a point in case, a comparison of Figure 7 with Figure 2B of [17] shows that, at a false positive rate of 10%, PHUNCTIONER achieves approximately 36% true positive rate (true positive rate≙sensitivity, false positive rate≙1−specificity). The GOdot method reaches 53% true positive rate at that false positive rate.

### Application to Structural Genomics Proteins

GOdot was applied to 500 query proteins corresponding to PDB entries labeled with unknown function and obtained by structural genomics initiatives. We analyzed the GOdot results for the subset of these proteins having four or more GO terms with a consensus combined score >1–10^−10^ (49 in total). For 13 of these proteins the predictions included at least one problematic GO term. In most of these cases the problematic GO term was annotated to protein neighbors that were multidomain proteins. These GO terms corresponded to the molecular function of a particular domain outside the region of sequence or structure similarity. Four additional proteins remain uncharacterized according to public annotation databases. The corresponding GOdot predictions were not necessarily incorrect, but they only included GO terms that were not very informative. Most of the GOdot predictions (32 proteins) were consistent with additional functional information that had been made available in the UniProt [38] database or in the literature.

Direct experimental evidence for the function annotation was usually not available for these proteins with consistent GOdot predictions. One case with experimental evidence is Cytochrome P450 from *Sulfolobus tokodaii* [39], PDB ID 1ue8. In other cases the structural model provides direct evidence for the molecular function, for instance if the model includes a ligand binding to the protein. The PH0226 protein from *Pyrococcus horikoshii* (PDB ID 1ve3) is such an example. The crystal structure includes the cofactor S-adenosyl-L-methionine (SAM) bound to the protein. The protein also shows significant structural similarity to other SAM-dependent methyltransferases, and is a member of the Methyltransferase homologous family, as identified by Pfam [40]. This evidence is consistent with the GOdot prediction of S-adenosylmethionine-dependent methyltransferase activity (GO∶0008757) with a combined consensus score of 1–10^−11^. This same query was used to illustrate the GOdot function prediction process in Figure 4. In other cases the available annotation is scarce and relies heavily on the detection of relationships to other proteins using either sequence or structure comparison methods. GOdot complements these approaches by providing an estimate for the function conservation given the extent of sequence and structure similarity.

**Figure 8:**
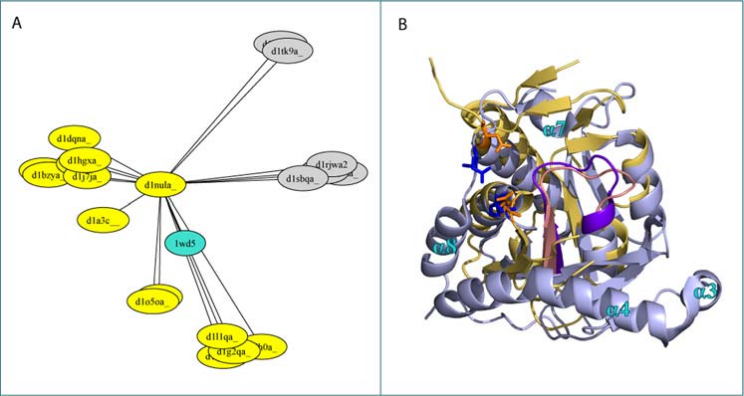
Query TT1426 (PDB 1wd5). A) Structural neighbors of hypothetical protein TT1426 (PDB 1wd5) according to TM-align. The image was generated by multidimensional scaling in the same way as Figure 4A. Proteins annotated with GO term GO:0016757 (glycosyltransferase activity) are colored yellow and they form a large group on the lower left, where the query is also located. The glycosyltransferase group is subdivided into subgroups. In general these subgroups are associated with different substrates, in particular adenine phosphoribosyltransferase (d1l1qa, d1g2qa), uracil phosphoribosyltransferase (d1o5oa, d1a3c), or xanthine/hypoxanthine/guanine phosphoribosyltransferases (d1nula, d1hgxa, d1dqna, d1j7ja, d1bzya). Proteins not annotated with GO∶0016757 are colored grey. They are less structurally related to the query than the glycosyltransferases, and accordingly they group separately on the right and top. B) Structural superposition of query TT1426 (PDB 1wd5 [41] in light blue) and the nearest neighbor, xanthine phosphoribosyltransferase (ASTRAL d1nula [45] in gold). The conserved 5-phosphoribosyl-1-pyrophosphate (PRPP)-binding motif characteristic of type I PRTases is colored pink in 1wd5, and violet in d1nula. Residues Arg32 and Lys56 in the query 1wd5 are shown in blue sticks. They are likely to be functionally relevant (involved in binding the pyrophosphate [41]). The structurally equivalent residues in the nearest neighbor are shown in orange. The structural differences in helices *α*3 and *α*4, as well as in the substrate binding C-terminal hood region (helices *α*7 and *α*8), indicates that they might have different substrates.

The hypothetical protein TT1426 from *Thermus thermophilus* provides an example of GOdot results complementing previous functional analysis. TT1426 has been identified in Pfam as a member of the Phosphoribosyl transferase domain family. The structure has been determined [41], PDB ID 1wd5, and predicted to be a phosphoribosyl transferase type I based on structural similarity to other proteins of the same family. GOdot predicts TT1426 to have a glycosyltransferase activity (GO∶0016757) with high reliability (combined consensus score is 1–5·10^−11^, as expected for a phosphoribosyl transferase. Figure 8A shows the structural relationships between the query and the structural neighbors according to TM, which are used to make GOdot predictions. The structural neighbors of the query are all glycosyltransferases, with structural subgroupings corresponding to distinct substrates. In Figure 8B, the structure of the query is compared to the nearest neighbor (a xanthine phosphoribosyltransferase). Both, the fold and the phosphoribosyl pyrophosphate-binding motif are conserved in the two proteins indicating that they share a phosphoribosyltransferase function. The differences in peripheral secondary structure elements indicate that they might have different substrates.

In summary, the manual inspection revealed 13 problematic cases (out of 49 proteins) for which a function was predicted falsely due to an invalid transfer of function from a multidomain protein. Four proteins could be neither confirmed nor refuted. For 32 proteins the GOdot predictions were manually confirmed with various other sequence or structure-based methods. See Table S1 for further details.

### Conclusions

We propose the GOdot method for predicting molecular function of proteins. The method uses functionally conserved regions as a new concept. These functional conservations are determined by statistical learning on a representative set of protein domains. Protein sequence and structure information of an unannotated protein are used as input to GOdot, which then predicts a list of GO terms. Each of the predicted GO terms has a reliability estimate attached which is computed based on the previously determined functionally conserved regions.

Both, the assessment using cross-validation on a representative dataset and the comparison with PHUNCTIONER [17] demonstrate that the analysis of functionally conserved regions is a powerful tool for function prediction: reference function predictors are considerably outperformed by the GOdot method. A high function conservation score is shown to indicate a high likelihood that a predicted GO term is correct. Consequently, function conservation scores can be used as reliability estimates within the prediction procedure.

To our knowledge, the GOdot method is the first approach that directly addresses the problem of estimating varying local function conservation in protein space with respect to different measures for protein similarity. For each similarity measure, each GO term and each protein domain in the representative training set, function conservation is captured with a logistic curve. The result is a large number of mutually intertwined and overlapping logistic curves. The set of logistic curves offers a new view on the relation between sequence and structure on the one hand and function on the other hand. We regard the analysis of functionally conserved regions as an important contribution to current function prediction efforts, and we expect forthcoming developments in this field to uncover more detailed insights into the sequence-structure-function space.

Local function conservation within protein space can be determined with respect to other protein similarity measures, such as shape or surface properties of protein binding sites, for example. The GOdot method can be easily extended to include other quantitative measures of protein similarity. For any new similarity measure one would simply perform an all-against-all comparison on the training set of proteins. Local function conservation can then be determined for that similarity measure. We are working on extending the GOdot method with new similarity measures to further improve its performance.

The GOdot method is available online as a web-server (http://godot.bioinf.mpi-inf.mpg.de), to which one can submit uncharacterized PDB structures. The method performs sequence and structure comparisons of the query protein to each entry from the representative set of protein domains. GO terms are predicted and function conservation scores are computed as reliability estimates. A ranked list of predicted GO terms is the output of the web-server.

## Methods

### The Functionally Annotated Protein Data Set

The analysis is based on a representative set of protein sequences and structures annotated with function data. We downloaded a set of 7290 protein domains with no more than 40% sequence identity from the ASTRAL compendium (version SCOP 1.69) [42]. These protein domains were assigned to the respective PDB structures. The PDB structures were mapped to UniProt sequences using the PDBSWS [43]. UniProt sequences were annotated with GO terms using the Gene Ontology Annotation (GOA) UniProt Gene association file (version 36.0) [33],[34]. We removed all domains having no GO annotation or being part of multidomain proteins according to SCOP. This representative set comprises 3449 protein domains annotated with 1806 distinct GO terms.

#### On GO levels

The GO vocabulary is structured as a directed acyclic graph (DAG). A GO term can have several parent terms. The annotation of a specific GO term to a protein then implies the annotation of all parent terms to that protein as well (this is referred to as the GO true path rule). As the GO vocabulary is organized as a DAG, a particular term can have several paths of different lengths to the root node; the term can occur on multiple levels of the ontology.

Performance comparisons across GO terms from different levels of specificity are hazardous. Therefore, in our studies we focus mostly on GO terms from level three, where the GO root is defined as being at level zero and GO ‘molecular function’ as level one. We consider a GO term to belong to level three, if it has any path of length three to the root node. An example of a level three GO term is ‘transferase activity’ (GO∶015972), which has the parent ‘catalytic activity’ (GO∶052747), which is in turn a direct child of ‘molecular function’ (GO∶161526).

### Learning Molecular Function from Sequence and Structure Data

Similarity between proteins is measured using different distance measures. We refer to observing a specific protein function consistently within a neighborhood of proteins in protein space as function conservation. We used different measures of similarity between proteins and describe a mathematical model for capturing function conservation. This model can be computed in a pre-processing step and later be used to predict protein function.

#### Computing similarities between proteins

For a pair of protein domains *p*,*r*, we compute similarities *sim*(*p*,*r*) using four different methods. The CE [36] and TM-align [37] programs compute structure-based similarity scores (*sim_CE_* and *sim_TM_*). Global profile (GP) and local profile (LP) alignments [35] capture the similarities (*sim_GP_* and *sim_LP_*) of the proteins' sequences as a whole or as the best partial match, respectively.

#### Fitting curves using logistic regression

We have determined conservation of molecular function with respect to the four similarity measures mentioned above. For each similarity measure *sim*, we apply the following training procedure to all protein domains in the dataset. Each protein domain *p* is annotated with a set *gt*(*p*) of molecular function GO terms. Let 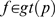 denote one of these terms. Note that, by the true path rule, *gt*(*p*) contains all of *f*'s parent terms.

For each term *f* annotated to a domain *p*, we determine conservation based on the occurrence of *f* among the nearest neighbors of domain *p*. The more neighbors of *p* have the same molecular function term *f* and the closer these neighbors are to *p*, the higher is the local conservation of *f* around *p*. We represent local function conservation using logistic regression as follows. Let *r*_1_,…*r_k_* denote the *k* nearest neighbors to *p* according to *sim*. In the experiments, we chose *k* = 200. Let *X* be the real-valued vector of similarities *X* = [*sim*(*p*,*r_1_*),…,*sim*(*p*,*r_k_*)]. Let *Y* denote the binary vector of observations describing for each of the nearest neighbors *r_i_*, whether *f* or more specific terms among its descendants are annotated to *r_i_*

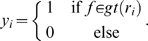



The logistic regression relates the similarities *X* between proteins and their neighbors to the log-odds that the respective neighbors are annotated with the same GO term (as indicated by *Y*). This relation is mathematically modeled by the logistic curve
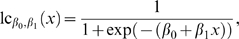
that is characterized by two parameters *β*_0_ and *β*_1_. Given *X* and *Y*, logistic regression yields *β*_0_ and *β*_1_. We fit such a logistic curve for each annotation of a GO term to each protein in the dataset. The multitude of logistic curves provides a view on the distribution of functional conservation.

### Predicting Protein Molecular Function

The GOdot method takes a query protein as input and predicts scores for a number of GO terms. For a query, we first predict scores representing the degree of GO function conservation. These scores are based on the local function conservation of the terms annotated to the query's nearest neighbors. The predicted scores are combined to account for multiple occurrences of related GO terms. Finally, ranking the GO terms according to the combined scores, the method produces a sorted list of GO terms.

Using logistic curves to estimate local function conservation.

A typical function prediction commences with a query protein *q* of unknown function. We identify *q*'s nearest neighbor with respect to the similarity measures, for example with *sim_CE_* as mentioned above. Let *x* = *sim_CE_*(*q*,*r*) be the similarity between *q* and the nearest neighbor *r*. The logistic curve previously computed for the neighbor *r* and one GO term *f* is used to estimate the likelihood of the GO term *f* occurring at similarity *x* to *r*. For a given similarity *x* and one GO term *f*, the *raw function conservation score* 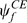 is defined as
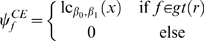
where *β*_0_ and *β*_1_ are the parameters representing the logistic curve for the particular GO term *f* attached to the particular nearest neighbor *r*. Thus, *ψ_f_* can be interpreted as estimated probability of *q* having the same GO term *f*, given a similarity *x* to the neighbor *r*. For the other similarity measures *sim_TM_*, *sim_GP_*, *sim_LP_*, the raw function scores are defined accordingly.

Combining raw function conservation scores along the GO graph structure.

For a query protein *q*, the different similarity measures point to potentially different nearest neighbors. These nearest neighbors are annotated with one or several GO terms. For each of these GO terms the raw function score provides an estimate of the likelihood that the transfer to the query is valid, at the given similarity. Thus, for a specific GO term *f*, we have four raw function scores attached to a protein, which we refer to as support 
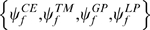
. As the GO terms are interconnected via the GO hierarchy, the support partially relates to each other and needs to be combined.

We merge several raw function conservation scores into one *combined function conservation score* per GO term. To this end, we propose the following score combination schemes which are applied to each GO term and incorporate the raw conservation scores of descendant GO terms. These combination schemes also ensure that GO terms obtain scores that are at least as high as those of their descendants.

**Figure 9:**
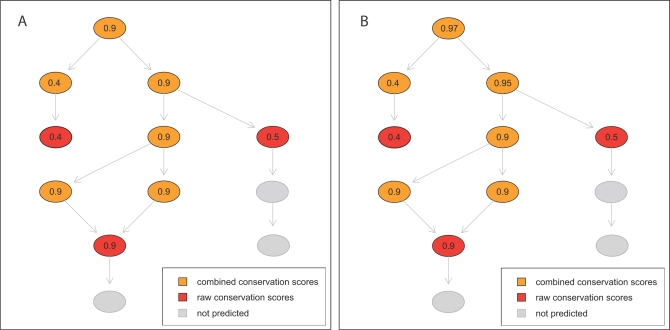
Selective and Consensus Combination Schemes. Examples of selective (A) and consensus (B) raw score combinations. (A) and (B) both show a subgraph of the full gene ontology. Raw function conservation scores were mapped to specific GO terms (red). We compute combined function conservation scores for more general GO terms (orange) using the selective and consensus combination schemes. Grey nodes indicate GO terms, that were not predicted by the method.

The *selective score combination* scheme computes the combined function conservation 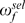 of a GO term *f* as the maximum raw function conservation score within the support of all its descendants *f*′ as follows:
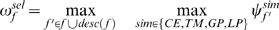
This selective score combination scheme is illustrated in Figure 9A.

The *consensus score combination* scheme computes the combined function conservation 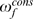. As mentioned before, the function conservation scores can be interpreted as probabilities. The probability of a GO term being correct is computed from the probabilities of the descendant GO terms being correct. The probability of a term being correct is one minus the probability that all descendant terms are incorrect. Assuming independence, the probability for all descendant terms being incorrect is the product of their individual probabilities for being incorrect. Consequently, we define the combined consensus function conservation score as
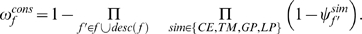
The combined consensus score depends on the number of descendants and the support observed for the descendants. High combined scores are caused by many descendants with high raw scores. The consensus score combination scheme is illustrated in Figure 9B.

Each of the combination schemes above produces one combined score per GO term. These combined scores are estimates of the reliability of the predicted GO terms. The GO terms predicted for one query are ranked with respect to the combined scores yielding a sorted list. We refer to a combination scheme producing such a list as *predictor*. The assessment of the predictors is described in the next section.

### Performance Assessment

We assess the GOdot method's performance by cross-validation. The selective and consensus predictors are compared to a baseline predictor using precision-recall graphs.

Cross-validation scheme.

We perform a leave-one-out cross-validation. Predictors are trained for each protein ignoring the annotations attached to that protein. In the Text S1 and Figure S1, we perform an additional significance analysis using ten-fold cross-validation.

#### Performance plots

We assess a predictor's performance with ROC plots. All GO terms that can be inferred from the nearest neighbors are considered and scored. An imaginary threshold is shifted from top to bottom over the list of ranked GO terms, treating all terms above the threshold as predicted. At each rank the number of true positives (TP≙correct GO terms predicted), false positives (FP≙incorrect GO terms predicted), true negatives (TN≙incorrect GO terms not predicted) and false negatives (FN≙correct GO terms not predicted) is counted. These counts are combined into the performance measures true positive rate and false positive rate. At each rank, the true positive rate is the fraction of true positive predictions from all positive samples and the false positive rate is the ratio of false positive predictions divided by the number of negative samples:
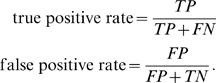
As a result we obtain pairs of true positive rate and false positive rate values for each rank in the list, yielding a ROC curve.

### Runtime Considerations

Predicting functions for a new query protein requires comparing the query to the set of representatives. Comparison of one protein to all 3449 protein domains in the set of representatives takes on average 4 hours for CE, 5 minutes for TM, and 2 minutes for profile alignment on a modern PC. With a compute cluster as back-end to the GOdot web-server, we provide answers typically within 20 to 60 minutes. In the training stage, establishing the protein space requires all-against-all comparisons, which is quite expensive (300 CPU days). When the distances are available, computing the logistic curves for 28774 annotations (of all-level GO terms to 3449 proteins) takes 9 minutes and is negligible in comparison.

## Supporting Information

Table S1 Details on Application to Structural Genomics Proteins. The table provides details on the 49 cases described in the paper, including PDB identifiers.
(0.06 MB PDF)

Text S1 Significance Analysis. Additional evaluation of the significance of the findings, based on an extended ROC-analysis / cross-validation
(0.06 MB PDF)

Figure S1 Performance assessment of consensus combination vs. selective combination. We use precision-recall graphs to compare the different predictors resulting from consensus score combination and selective score combination with predictors employing mere protein similarity measures and a background predictor. The plot is based on the cross-validation results, each curve describing the median performance of one distinct predictor. The boxes indicate 25% and 75% quantiles, the whiskers represent the maximum deviation from the median. The predictors employing protein similarity measures only, have a performance worse than the background predictor for very low recall rates. For very similar proteins, GO terms are predicted as likely, regardless of their level within the GO hierarchy. This leads to false terms predicted as very likely and thus to a precision of below 1 for recall 0.
(0.28 MB PDF)

Figure S2 Assessing similarity based inference on the high-quality data set. We entirely repeated the estimates and calculations performed for the high-coverage data set in the main manuscript on a high-quality data set. This high-quality data set is restricted to annotations that stem traceably from literature (evidence code TAS) or from direct experiments (evidence codes IDA, IEP, IGI, IMP, IPI), leaving 945 proteins with curated experimental annotations. This figure corresponds to Figure 1 in the main paper, with the evaluation performed on high-quality annotations.
(0.17 MB PDF)
