# Supplementary material for: Local Function Conservation in Sequence and Structure Space
Source: PLoS Comput Biol. 2008 Jul 4;4(7):e1000105. doi: 10.1371/journal.pcbi.1000105 (PMC2427199; doi:10.1371/journal.pcbi.1000105)
Supplement: Table S1 — Details on Application to Structural Genomics Proteins. The table provides details on the 49 cases described in the paper, including PDB identifiers. (0.06 MB PDF) [file pcbi.1000105.s001.pdf]

Local Function Conservation in Sequence and Structure Space: Supporting Table S1

Nils Weinhold[1], Oliver Sander[1], Francisco S. Domingues[1], Thomas Lengauer[1], Ingolf Sommer*[1]

[1]Max Planck Institute for Informatics, Stuhlsatzenhausweg 85, 66123 Saarbrücken, Germany

Email: Nils Weinhold - weinhold@mpi-inf.mpg.de; Oliver Sander - osander@mpi-inf.mpg.de; Francisco S. Domingues - doming@mpi-inf.mpg.de; Thomas Lengauer - lengauer@mpi-inf.mpg.de; Ingolf Sommer - sommer@mpi-inf.mpg.de;

*Corresponding author

Application to Structural Genomics Proteins

**32**	**Consistent Predictions**	
22	Correct prediction for previously characterized proteins	1UE8, 1K26, 1JRK, 1K2E, 1JN1, 1RTT, 1RXD, 1UMJ, 1V99, 1J2V, 1KR4, 1VHF, 1P1L, 1V6H, 1NZA, 1FL9, 1V9B, 1RZW, 1TXL, 1NMN, 1NPD, 1WI6
8	Prediction agrees with sequence based methods (PFAM & others)	1SQH, 1WDT, 1VP8, 1RFE, 1W9A, 1TQX, 1VE3, 1WD5
2	Correct prediction for at least one functional term, but not all (prediction is not complete)	1O4T, 1WIG
**13**	**Problematic Predictions**	
10	Incorrect prediction by transferring function from domain that was not aligned in a multidomain hit	1LPL, 1TOV, 1WHK, 1WHJ, 1VG5, 1WHR, 1VEG, 1WGN, 1WIV, 1VEK
2	Prediction for uncharacterized proteins, does not match additional data	1VPV, 1TP6
1	Incorrect prediction for characterized protein	1RZ2
**4**	**Inconclusive Predictions**	
4	Inconclusive prediction for uncharacterized proteins (besides correct prediction of metal binding)	1WIL, 1SFN, 1VJ2, 1V70
