# Supplementary material for: Local Function Conservation in Sequence and Structure Space
Source: PLoS Comput Biol. 2008 Jul 4;4(7):e1000105. doi: 10.1371/journal.pcbi.1000105 (PMC2427199; doi:10.1371/journal.pcbi.1000105)
Supplement: Text S1 — Significance Analysis. Additional evaluation of the significance of the findings, based on an extended ROC-analysis / cross-validation (0.06 MB PDF) [file pcbi.1000105.s002.pdf]

Local Function Conservation in Sequence and Structure Space: Supporting Text S1

Nils Weinhold[1], Oliver Sander[1], Francisco S. Domingues[1], Thomas Lengauer[1], Ingolf Sommer*[1]

[1]Max Planck Institute for Informatics, Stuhlsatzenhausweg 85, 66123 Saarbrücken, Germany

Email: Nils Weinhold - weinhold@mpi-inf.mpg.de; Oliver Sander - osander@mpi-inf.mpg.de; Francisco S. Domingues - doming@mpi-inf.mpg.de; Thomas Lengauer - lengauer@mpi-inf.mpg.de; Ingolf Sommer - sommer@mpi-inf.mpg.de;

*Corresponding author

Performance Assessment

While in the main manuscript, we analyze at the differences in performance of various function predictors, here we analyze the significance of these differences. We assess the significance of the differences in combining the scores within the Godot method by ten-fold cross-validation. The selective and consensus predictors are compared to baseline predictors using precision-recall graphs. The results are summarized in a precision-recall graph in supporting Figure S1. An optimal predictor's curve would pass through the point (1,1) in the precision-recall graph. The error bars attached to the precision-recall curves in Figure 5 indicate that the differences discussed here are significant.

Cross-validation Scheme

We perform a ten-fold cross-validation as follows. The 3449 protein domains are partitioned into ten equally sized subsamples. Predictors are trained on nine subsamples, the remaining subsample is used for testing. Each subsample is used for testing once. This performance estimate tends to underestimate performance as each predictor employs only 90% of the 3449 proteins for training.

Performance Plot

We assess a predictor's performance with a precision-recall plot. An imaginary line is shifted from top to bottom over the list of ranked GO terms, treating all terms above the line as predicted. At each rank the number of true positives (TP $\widehat{=}$ correct GO terms predicted), false positives (FP $\widehat{=}$ incorrect GO terms predicted), true negatives (TN $\widehat{=}$ incorrect GO terms not predicted) and false negatives (FN $\widehat{=}$ correct GO terms not predicted) is counted. These counts are combined into the performance measures precision and recall. At each rank, precision is the fraction of all predictions that are correct and recall is the fraction of all correct GO terms that were predicted:

$$\begin{aligned} precision &= \frac{TP}{TP+FP} \\ recall &= \frac{TP}{TP+FN}. \end{aligned}$$

As a result we obtain pairs of precision and recall values for each rank in the list, yielding a precision-recall curve. During the ten-fold cross-validation procedure we calculate one such curve for each protein domain. The curves are averaged fold-wise, producing 10 averaged curves, one per fold. Each curve in Figure 5 displays the median precision of these 10 curves at 100 equidistant sampling points along the recall axis. An optimal predictor's curve would pass through (1,1).
