# Supplementary figures and images for: Local Function Conservation in Sequence and Structure Space
Source: PLoS Comput Biol. 2008 Jul 4;4(7):e1000105. doi: 10.1371/journal.pcbi.1000105 (PMC2427199; doi:10.1371/journal.pcbi.1000105)

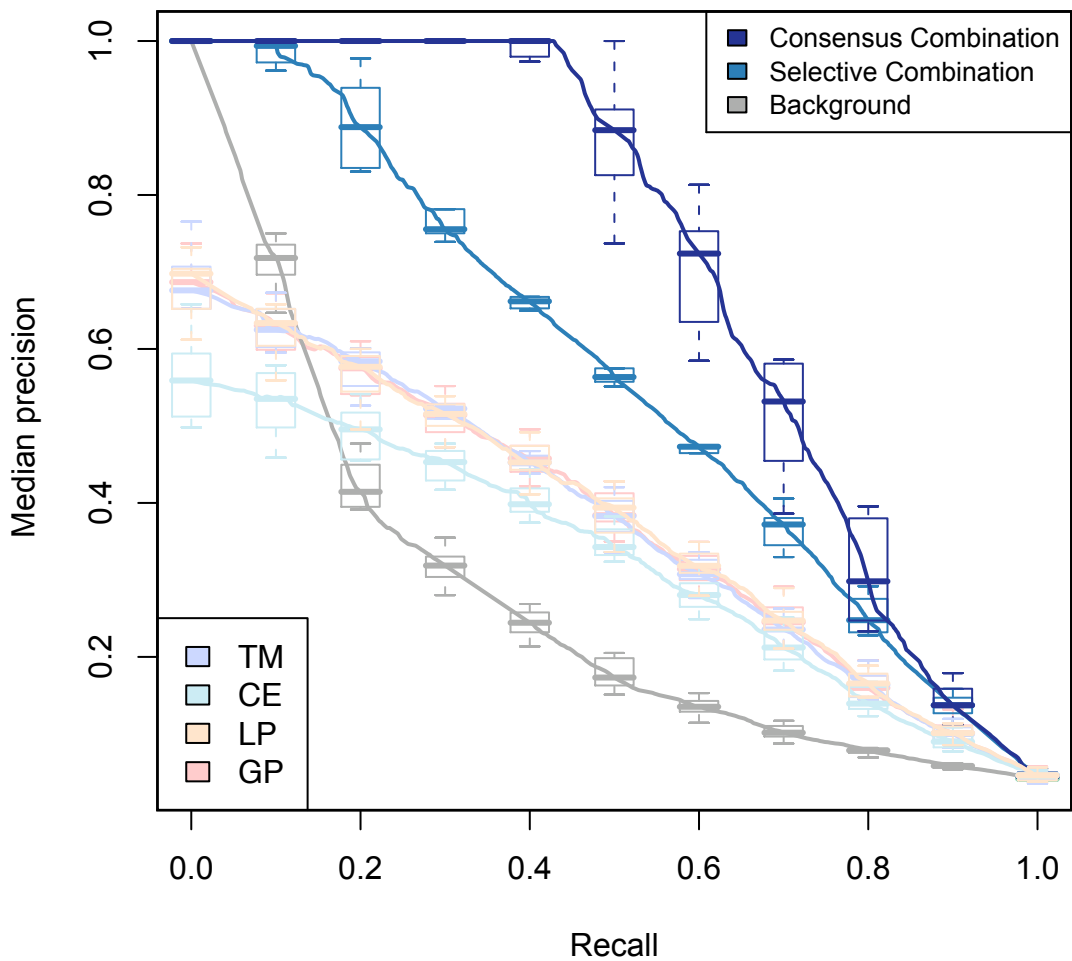

Supplement: Figure S1 — Performance assessment of consensus combination vs. selective combination. We use precision-recall graphs to compare the different predictors resulting from consensus score combination and selective score combination with predictors employing mere protein similarity measures and a background predictor. The plot is based on the cross-validation results, each curve describing the median performance of one distinct predictor. The boxes indicate 25% and 75% quantiles, the whiskers represent the maximum deviation from the median. The predictors employing protein similarity measures only, have a performance worse than the background predictor for very low recall rates. For very similar proteins, GO terms are predicted as likely, regardless of their level within the GO hierarchy. This leads to false terms predicted as very likely and thus to a precision of below 1 for recall 0. (0.28 MB PDF) [file pcbi.1000105.s003.pdf]

A

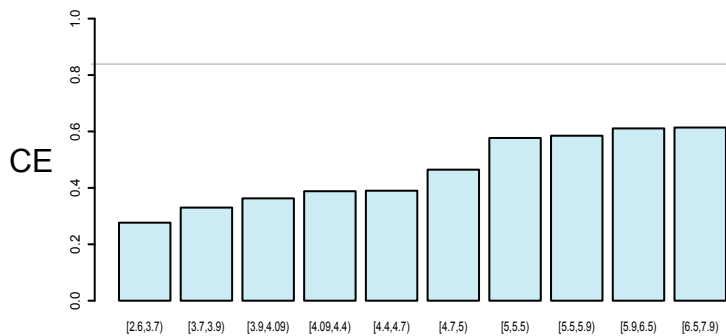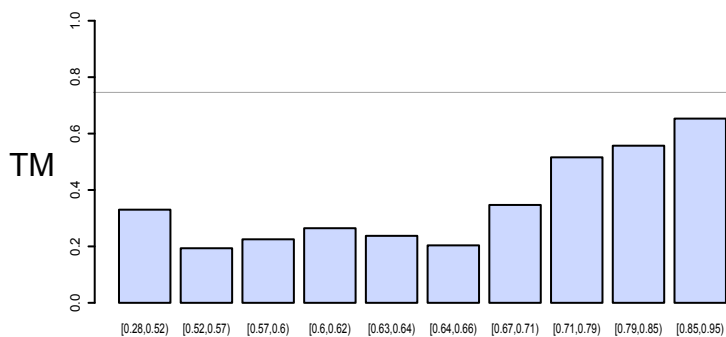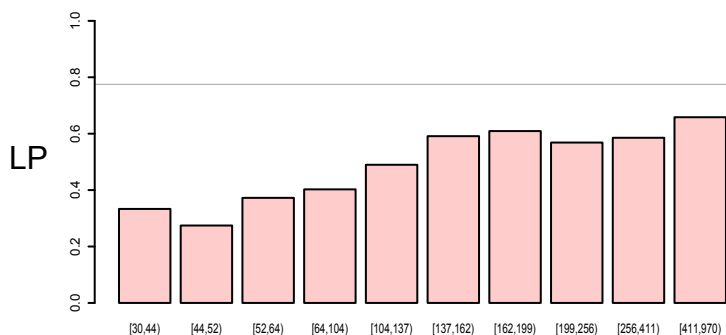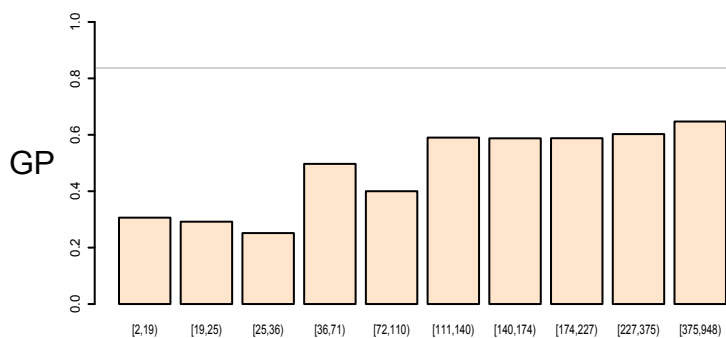

B

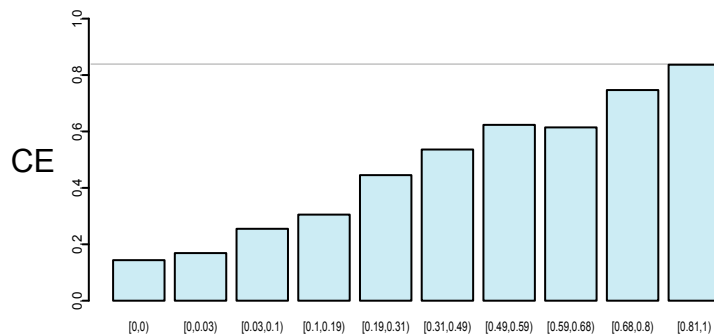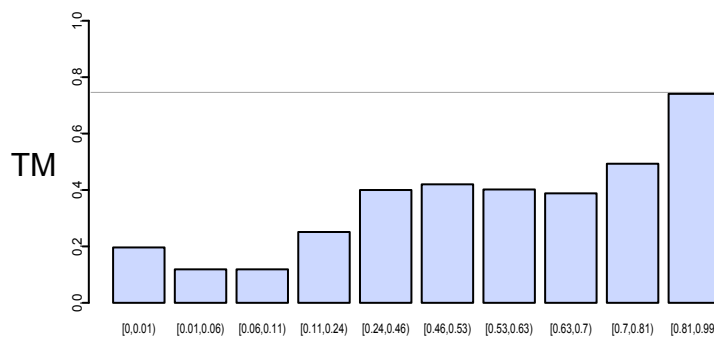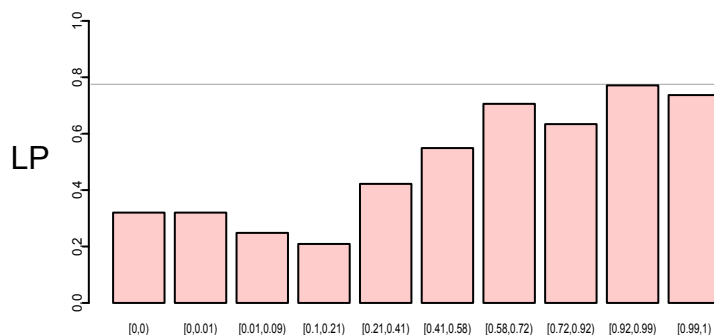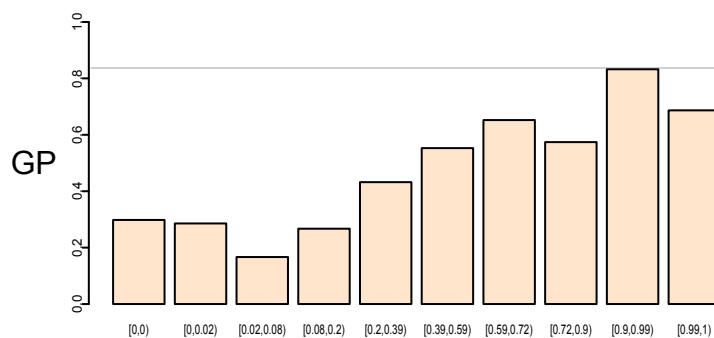

Supplement: Figure S2 — Assessing similarity based inference on the high-quality data set. We entirely repeated the estimates and calculations performed for the high-coverage data set in the main manuscript on a high-quality data set. This high-quality data set is restricted to annotations that stem traceably from literature (evidence code TAS) or from direct experiments (evidence codes IDA, IEP, IGI, IMP, IPI), leaving 945 proteins with curated experimental annotations. This figure corresponds to Figure 1 in the main paper, with the evaluation performed on high-quality annotations. (0.17 MB PDF) [file pcbi.1000105.s004.pdf]
